# Sword Bean (*Canavalia gladiata*) Pods Induce Differentiation in MC3T3-E1 Osteoblast Cells by Activating the BMP2/SMAD/RUNX2 Pathway

**DOI:** 10.3390/nu15204372

**Published:** 2023-10-16

**Authors:** Yu Jin Hwang, Hye-Jeong Hwang, Hyunseo Go, NaYeong Park, Kyung-A Hwang

**Affiliations:** 1 Department of Agrofood Resources, National Institute of Agricultural Sciences, Rural Development Administration, Wanju-gun 55365, Republic of Korea; yjhwang1022@korea.kr (Y.J.H.); hjh1027@korea.kr (H.-J.H.); gustj1122@korea.kr (H.G.);; 2 Department of Food and Biotechnology, Korea University, Sejong City 30019, Republic of Korea

**Keywords:** sword bean pods, MC3T3-E1 cells, osteoblast, BMP2/SMAD/RUNX2 pathway

## Abstract

Sword bean (SB) contains various phytochemicals, such as flavonoids, tannins, saponins, and terpenoids. Although the evaluation of its potential functions, including antioxidant, anti-obesity, anti-inflammatory, liver protection, and antiangiogenic activities, has been widely reported, research on their use in osteoporosis prevention is insufficient. Furthermore, while various studies are conducted on SB, research on sword bean pods (SBP) is not yet active, and little is known about it. Therefore, this study investigated the effects of promoting osteoblast differentiation of MC3T3-E1 cells using SB and SBP extracts and their mechanisms. We show that SBP extracts increase osteoblast proliferation, mineralization-activated alkaline phosphatase (ALP), and collagen synthesis activities. Additionally, treatment with SBP extract increased the expression of markers related to osteoblast differentiation, such as ALP, SPARC, RUNX2, COL-I, BMP2, OCN, and OPN. It was confirmed that SBP induces differentiation by activating the BMP2/SMAD/RUNX2 pathway. We also show that SBP is more effective than SB, and SBP may be useful in assimilating bone minerals and preventing osteoporosis.

## 1. Introduction

Skeletal homeostasis is maintained through constant bone formation and resorption. The mechanism of bone absorption and bone formation in the skeletal system is regulated by the balance between osteoblasts. However, in elderly or postmenopausal women, an imbalance occurs due to excessive bone resorption compared to bone formation, resulting in skeletal disorders such as osteoporosis [1]. Osteoporosis is characterized by the deterioration of bone tissue structure and low bone density, leading to fragility and bone fractures. It is considered the most common major bone disease worldwide [2]. As aging accelerates, the prevention and treatment of osteoporosis, which emerges as a major health problem in old age, needs urgent attention in the medical field.

Since the proper function of osteoblasts involves increasing growth and proliferation or stimulating differentiation, it is necessary to develop a material that can stimulate osteoblast differentiation. The differentiation of osteoblasts is controlled by various enzymes and proteins. Among the proteins involved in osteogenesis, bone morphogenetic protein 2 (BMP2), a crucial member of the transforming growth factor-β superfamily, is pivotal in regulating osteoblast differentiation [3,4,5]. Several downstream signaling pathways have been reported to be stimulated upon the binding of BMP2 to its ligand/receptors. BMP2 signaling leads to the phosphorylation of SMAD proteins, such as SMAD1/5/9, which controls many transcription factors of osteoblast differentiation markers [4,5]. Bone formation-related factors, such as alkaline phosphatase (ALP) activity, type I collagen (COL-I), osteocalcin (OCN), and osteopontin (OPN), are vital differentiation markers of osteoblasts [6], and their expressions are controlled by the major transcription factor, Runt-related Gene 2 (RUNX2). RUNX2 is a critical transcription factor required for osteogenesis, playing a vital role in activating genes responsible for osteoblast differentiation. Specifically, by binding to a cis-acting element within the promoter region of OCN, Runx2 initiates the expression of this marker gene. Studies conducted in the past have demonstrated that RUNX2 knockout mice exhibit decreased chondrocyte maturation and a failure to form bones [7]. Additionally, the expression of secreted protein acidic and cysteine-rich (SPARC, also called osteonectin) is also affected by RUNX2. SPARC is the most abundant non-collagenous protein in the bone matrix and is secreted by bone osteoblasts during bone formation [8,9]. It is associated with the expression of OCN, OPN, and growth factors involved in bone formation. SPARC commits cells to the osteoblast lineage by inhibiting their differentiation into adipocytes [10]. Several studies have suggested that activation of BMP2/SMAD and RUNX2 signals is involved in osteoblast differentiation [11,12].

Most drugs currently used to prevent and treat osteoporosis are bone resorption inhibitors, which cannot restore lost bone. Therefore, these drugs are difficult to use for preventing and treating osteoporosis that has already progressed [13]. Therefore, active research is being conducted on natural therapeutic compounds that promote bone formation through stimulation of osteoblast differentiation. Currently, natural plant-derived phytoestrogen has been reported to be effective in treating menopausal osteoporosis [14,15]. Additionally, several studies have reported the effects of promoting osteoblast differentiation and bone formation from various natural products. As a result of previously reported studies, *Cissus quadrangularis* was found to promote osteoblast differentiation through increased ALP activity and matrix mineralization [16]. Similarly, *Dendropanax morbifera* increased downstream ALP, OPN, OCN, and RUNX2 via BMP2/p38 MAPK/JNK and SMAD1/5/9 signaling pathways [17]. In addition, *Panax ginseng* [18] and *paeoniflorin* [19] have also been shown to promote osteoblast differentiation and bone formation.

Sword bean (SB; *Canavalia gladiata*) grows year-round and is native to tropical Asia, Central America, and Africa. With a history of use as a medicinal plant in Asia spanning thousands of years, dried sword beans were also utilized as a food source in southwestern and Central America. SB has three phenotypes, red, black, and white. The three types of SB contain various components such as gallic acid, methyl gallate, 1,6-di-O-galloyl-β-D-glucopyranoside, β-sitosterol, lupeol, and d-tocopherol. In particular, both the soluble and bound fractions of black and red SB are rich in gallic acid and ellagic acid. It has been confirmed that these ingredients are present at higher levels in black and red SB compared to white SB [20,21]. Although black and red SB are richer in bioactive components than white SB, white SB is mainly cultivated in Korea, and white SB and its pods are registered as food ingredients with the Ministry of Food and Drug Safety. Therefore, many researchers in Korea conducted various studies to evaluate the potential of white SB as a functional material. Recent studies have also shown that SB has various physiological activities, including antioxidant [21], anti-inflammatory [22], improved hematopoietic dilatation [23], liver protection, and antiangiogenic activity [24]. Their functionality is due to the various components present in SB. Various studies have reported that white beans of SB are rich in phytochemicals, such as alkaloids, saponins, flavonoids, terpenoids, steroids and tannins, and nutrients, including proteins, carbohydrates, minerals, and vitamins [25]. It also contains hemagglutinin, canavanine, urease, and canavalia gibberellin I and II.

In addition to SB beans, various parts such as pods, roots, and stems have been used in folk remedies for dysentery, nausea, hemorrhoids, sinusitis, backaches, and obesity [26,27]. In particular, although SB pods (SBP) contain as many functional ingredients as SB, not much research has been conducted on the functionality of SBP. According to our previous study, nine free phenolic acids were detected in SBP [22]. Among them, pyrogallol and sinapic acid were included in the highest abundance. Pyrogallol is contained in tea and coffee beans and has physiological activities such as antioxidant [28] and antimicrobial [29] properties. Sinapic acid is found in vegetables, cereals, and oilseed crops and has been reported to have the potential to improve various pathological conditions, including inflammation [30], diabetes [31], and neurodegeneration [32].

It has been reported that ingredients such as proline [33], citric acid [34], sinapic acid [35], and tannin [36] contained in SB or SBP promote bone health by differentiating osteoblasts. However, no research has yet been conducted on the osteoblast activity of SB or SBP.

Thus, in this study, we investigated the effects of SB and SBP extracts in promoting osteoblast differentiation on MC3T3-E1 cells and conducted a comprehensive analysis of their functionality.

## 2. Materials and Methods

### 2.1. Reagents

The α-Minimum Essential Medium (αMEM), penicillin/streptomycin antibiotics (P/S), phosphate-buffered saline (PBS), and trypsin were purchased from Gibco (Gaithersburg, MD, USA). The following reagents were purchased from Sigma-Aldrich (St. Louis, MO, USA): Dexamethasone, L-ascorbic acid, β-glycerophosphate, dimethyl sulfoxide (DMSO), 3-(4,5-dimethylthiazol-2-yl)-2,5-diphenyl tetrazolium bromide(MTT), NP-40, triton X-100, sodium deoxycholate, sodium chloride, EDTA, p-nitrophenyl phosphate(p-NPP), Trizma base, MgCl_2_, sodium hydroxide, paraformaldehyde, alizarin red S, cetylpyridinium chloride, picro-sirius red solution, acetic acid, ethanol, ortho-cresolphthalein complexone, and 3-mercaptoethanol. Polyvinylidene difluoride (PVDF) membranes, sodium dodecyl sulfate-polyacrylamide gels for SDS-PAGE, and a Chemi-doc image detector (Chemi-Doc XRS+ System) were purchased from Bio-Rad (Hercules, CA, USA). Antibodies against BMP2, Runx2, p38, p-p38, Smad 1/5/9, p-Smad 1/5/9, JNK, p-JNK, ERK, and p-ERK were purchased from Abcam (Cambridge, MA, USA). Fetal bovine serum (FBS), bicinchoninic acid (BCA) protein assay reagent, and anti-rabbit IgG horseradish peroxidase-conjugated antibodies were purchased from GenDEPOT (Barker, TX, USA).

### 2.2. Preparation of Sample Extract

SB and SBP were purchased from domestically grown sources in Hwasun, Korea (Figure S1). After washing with water to remove impurities, SB and SBP were ground, dried for 8 h in hot air at 50 °C (Jeil Machinery Co., Ltd., Icheon, Republic of Korea), and pulverized using a grinder (IKA, M20, Staufen, Germany). The ground SB and SBP were mixed with 30% ethanol and stirred at 80 °C for 8 h. After primary extraction, the remaining solvent was used for secondary extraction at 80 °C for 4 h. After obtaining the extracts, they were filtered through a filter paper, concentrated using a vacuum concentrator (R-100; Doo Young High Technology, Seoul, Republic of Korea), and subjected to freeze-drying. The extraction yield of SB and SBP after lyophilization was 16.1% and 16.6%, respectively. Finally, the lyophilized samples were stored at −20 °C for future use.

### 2.3. Cell Culture and Differentiation

MC3T3-E1, the murine pre-osteoblast cell line, was purchased from the American Type Culture Collection (ATCC, Manassas, VA, USA). MC3T3-E1 cells were maintained in αMEM containing 10% FBS and 1% P/S in an incubator with 5% CO_2_ at 37 °C. The medium was changed twice a week, and when the cells reached 80–90% confluence, they were detached with 0.25% trypsin and plated for experiments. To induce osteogenic differentiation, cells were grown in differentiation media supplemented with 50 µg/mL L-ascorbic acid, 10 mM β-glycerophosphate, and 10 nM dexamethasone in α MEM culture media one day after seeding. The existing differentiation medium was discarded every three days and replaced with a fresh differentiation medium.

### 2.4. Cell Viability

The effects of SB and SBP on MC3T3-E1 cell viability for evaluating cell toxicity and cell proliferation were tested by the MTT assay. MC3T3-E1 cells were plated on 96-well plates at 5 × 10^3^ cells/well in an incubator with 5% CO_2_ at 37 °C. The cells were subjected to various concentrations of SB and SBP extracts after incubating for 24 h. The treatment lasted for 24, 48, and 72 h. After the sample treatment, 5 mg/mL of MTT reagent was added to each well and incubated for 4 h. The formazan crystals were dissolved in DMSO, and the absorbance was measured at 540 nm using a SpectraMax M5 (Molecular Devices, LLC, Sunnyvale, CA, USA) after discarding the medium.

### 2.5. ALP Activity

MC3T3-E1 cells were seeded in 24-well plates and incubated for 24 h in an incubator with 5% CO_2_ at 37 °C. After 24 h of incubation, the medium was replaced with differentiation media to induce osteoblastic differentiation. The experimental groups were treated with different concentrations of SB and SBP for 7 days, and the medium was replaced every 3 days. After 7 days of incubation, cells were washed with PBS and harvested in lysis buffer, and the supernatants were used for ALP analysis. For the activity of alkaline phosphatase analysis, sample or standard (4-nitrophenol) and 1M Trizma base buffer solution (pH 10) containing 5 mM MgCl_2_ and p-nitrophenyl phosphate were added in a 96-well plate. The mixture was incubated at 37 °C for 1 h, and the reaction was stopped by adding 0.3 N NaOH in each well. The ALP activity was measured using a microplate reader (SpectraMax M5; Molecular Devices, LLC, Sunnyvale, CA, USA) at 405 nm and normalized to the protein contents.

### 2.6. Collagen Synthesizing Activity

To evaluate the collagen synthesis-inducing activity of SB and SBP in MC3T3-E1 cells, collagen histochemistry was examined by Picro-Sirius Red (PSR) staining. Cells were seeded in 24-well plates and incubated for 24 h at 37 °C in the presence of 5% CO_2_. After 24 h of incubation, the medium was replaced with the differentiation media to induce osteoblastic differentiation and treated with different concentrations of SB and SBP for 14 days. The medium was replaced every 3 days. Before staining, cells were washed with PBS and fixed with 4% paraformaldehyde for 30 min. Fixed cells were stained with 0.1% PSR for 1 h at room temperature. Cells were rinsed with acidified water (0.5% acetic acid) and dehydrated with serial ethanol washes in the order of 70%, 90%, and 100% for 5 min each. PSR images were analyzed using a light microscope (Leica Microsystems, Wetzlar, Germany). After imaging, the stain was dissolved in 0.1 N NaOH, and the absorbance was measured at 550 nm using a microplate reader.

### 2.7. Mineralization Level Measurement

The mineralization level was assessed through alizarin red S (ARS) staining, followed by quantitative analysis. MC3T3-E1 cells were seeded onto a 24-well plate and induced to differentiate for 14 days while being treated with SB and SBP. Following this, the cells were washed with PBS and then fixed with 4% paraformaldehyde for 30 min. After another wash with PBS, the cells were stained with a 40 mM solution of ARS (pH 4.3) for 10 min. Next, the cells were rinsed with distilled water and left to air dry naturally. The images of stained cells were photographed using a light microscope (Leica Microsystems, Wetzlar, Germany). To measure the extent of matrix mineralization, cells were stained with ARS and dissolved in a solution containing 10% cetylpyridinium chloride in PBS (pH 7.0) for 15 min. The absorbance was then measured using a microplate reader set to 562 nm.

### 2.8. Real-Time Polymerase Chain Reaction (RT-PCR) 

MC3T3-E1 cells were incubated in 6-well plates, allowed to differentiate for either 7 or 14 days, and then treated with SB and SBP extracts. Following incubation, the cells were washed with PBS, and RNA was extracted using the RNeasy Plus Mini Kit (manufactured by Qiagen, Valencia, CA, USA). The extracted RNA was then used to synthesize cDNA, according to the manufacturer’s instructions, using M-MLV Reverse Transcriptase (manufactured by Promega, Madison, WI, USA). Next, the synthesized cDNA was used for RT-PCR, employing the amfiSure qGreen Q-PCR Master Mix (manufactured by genDEPOT) and the Qiagen RotorGene Q real-time PCR machine. The target gene was amplified using a temperature cycling protocol consisting of denaturation at 95 °C for 15 s, annealing at 57 °C for 20 s, and extension at 72 °C for 30 s. To determine the relative levels of mRNA, the expression levels of the target gene were normalized to those of the housekeeping gene, glyceraldehyde 3-phosphate dehydrogenase (GAPDH). The mouse primers used for the experiment can be found in Table 1.

### 2.9. Western Blot Analysis

MC3T3-E1 cells were seeded in 6-well plates, differentiated for 7 or 14 days, and treated with SB and SBP extracts in an incubator with 5% CO_2_ at 37 °C. After incubation, cells were harvested on ice with an analytical buffer and centrifuged at 4 °C for 10 min at 12,000× *g* to separate the proteins. After quantifying the protein through BCA protein analysis, 10 μg of protein were separated by 4% to 20% SDS-PAGE (Sodium Dodecyl Sulfate-Polyacrylamide Gel Electrophoresis) and transferred to PVDF membranes. The transferred membranes were blocked with 5% (*w/v*) skim milk in TBS-T buffer for 1 h at room temperature, followed by overnight incubation at 4 °C with primary antibodies against GAPDH, OCN, OPN, COL1, BMP2, Runx2, Smad1/5/9, p-Smad1/5/9. After treating the membrane, it was washed and incubated with secondary antibodies for 1 h at room temperature. The bands were then visualized using an enhanced chemiluminescence reagent (Thermo, Rockford, IL, USA) and captured using a Chemi-Doc image detector. The relative protein levels were quantified using the ImageJ program (ver. 1.53e, NIH, Bethesda, MD, USA).

### 2.10. Statistical Analysis

The statistical program SPSS (version v25.0, SPSS, Chicago, IL, USA) was used to analyze the experiment. All experiments were repeated three times. Data are expressed as the means ± standard deviation (SD). Student’s *t*-test was used to analyze the difference between the experimental and control groups, and a *p*-value less than 0.05 was considered statistically significant.

## 3. Results

### 3.1. Effects of SB and SBP on Osteoblastic Cell Viability

**Figure 1:**
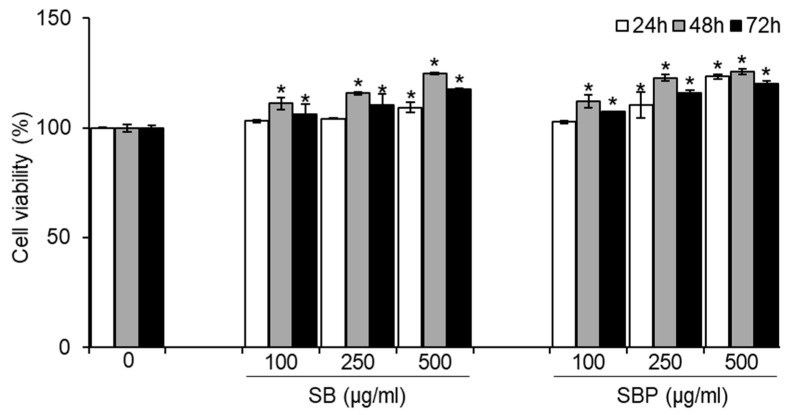
Effects of SB and SBP on MC3T3–E1 cell viability. Cells were treated with different concentrations (100, 250, 500 μg/mL) of SB and SBP extract for 24, 48, and 72 h. Cell viability was determined by MTT assay. Data are expressed as mean ± SD. * *p* < 0.05 compared to the sample-untreated group by Student’s *t*-test. SB, Sword bean; SBP, Sword bean pod.

To determine whether SB and SBP induce osteoblastic differentiation in MC3T3–E1 cells, their effect on cell growth was first evaluated. Cells were treated with various concentrations of SB and SBP (100, 250, and 500 μg/mL) for 24, 48, and 72 h, and cell viability was measured using the MTT assay. As shown in Figure 1, SB and SBP showed cell viability of more than 100% in pre-osteoblastic MC3T3–E1 cells up to 500 μg/mL, showing no cytotoxicity. In addition, SB and SBP treatment groups in 250 and 500 μg/mL showed significantly higher cell viability than the control group. Therefore, 100, 250, and 500 μg/mL concentrations were selected and used in subsequent experiments owing to their cell proliferation ability without causing cytotoxicity.

### 3.2. SB and SBP Induce ALP Activity in MC3T3–E1 Cells

**Figure 2:**
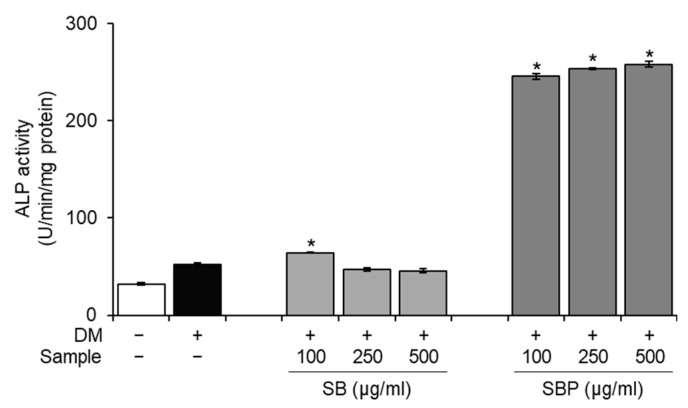
Effects of SB and SBP on alkaline phosphatase (ALP) activity in MC3T3–E1 cells. Cells were cultured with different concentrations (100, 250, 500 μg/mL) of SB and SBP extract for 7 days. Data are expressed as mean ± SD. * *p* < 0.05 compared to the DM only-treated group by Student’s *t*-test. DM, differentiation media; SB, Sword bean; SBP, Sword bean pod.

ALP, alkaline phosphatase, is a crucial phenotypic marker of osteogenesis and an essential enzyme during the early stages of osteoblastic differentiation. To evaluate whether SB and SBP stimulate osteoblastic differentiation, ALP activity was measured after incubation with or without SB and SBP for 7 days. It was confirmed that the ALP activity of the differentiated control group increased significantly compared to the non-differentiated control group, and the differentiation was appropriately induced. The SBP treatment group showed significantly higher ALP activity in all treated concentrations. Particularly at the highest concentration (500 μg/mL), ALP activity enhanced 5-fold compared to the differentiated control group (Figure 2).

### 3.3. Effects of SB and SBP on Intracellular Collagen Synthesize Activity

**Figure 3:**
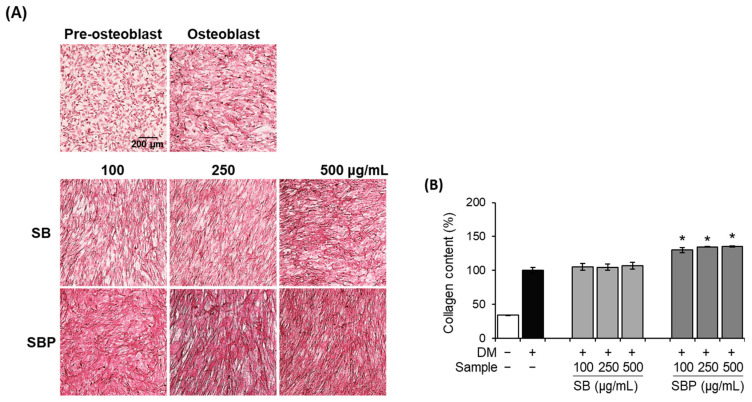
Effects of SB and SBP extracts on collagen synthesize activity in MC3T3–E1 cells. Cells were treated with or without differentiation media, with extracts at a concentration ranging from 100 to 500 μg/mL for 14 days. (**A**) Picro–sirius red (PSR) staining was performed and visualized by microscopy (×100 magnification). (**B**) Collagen content was evaluated by measuring absorbance at 550 nm. Values are expressed as mean ± SD. * *p* < 0.05 compared to the DM only-treated group by Student’s *t*-test. DM, differentiation media; SB, Sword bean; SBP, Sword bean pod.

Collagen content was evaluated histochemically using PSR staining to identify the effects of SB and SBP on collagen synthesis. Staining of collagen via PSR is predominant for types I and III, and a red color indicates more mature/well-packed/organized collagen. The microscopic images and the quantified results after staining are shown in Figure 3. The differentiated control group and SB- and SBP-treated groups showed red-stained collagen, unlike negative control. SB treatment group showed no significant increase. On the other hand, the SBP group showed a significant increase in collagen production in a concentration-dependent manner. In addition, the highest SBP concentration showed 113.5% collagen content against the differentiated control group. Through these results, it was confirmed that SBP has superior collagen synthesis ability than SB.

### 3.4. Effects of SB and SBP on Mineralization

**Figure 4:**
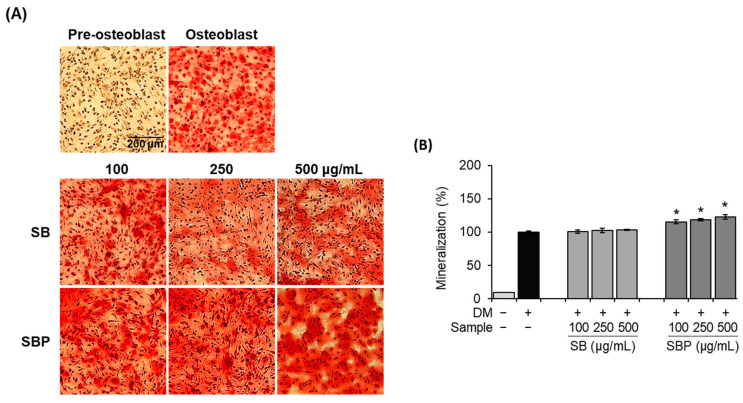
Effects of SB and SBP extracts on mineralization in MC3T3–E1 cells. Cells were treated with or without differentiation media with extracts at concentrations ranging from 100 to 500 μg/mL for 14 days. (**A**) Alizarin red staining (ARS) was performed and visualized by microscopy (×100 magnification). (**B**) Mineralization (%) was evaluated by measuring absorbance at 562 nm. Values are expressed as mean ± SD. * *p* < 0.05 compared to the DM only-treated group by Student’s *t*-test. DM, differentiation media; SB, Sword bean; SBP, Sword bean pod.

In general, bone formation requires proliferation, substrate maturity, and mineralization. Along with ALP and collagen synthesis activities, mineralization is essential for osteoblastic differentiation. To investigate the effects of SB and SBP on bone mineralization, both histochemical and quantitative assays were conducted using ARS. It was confirmed that the osteoblast group and SB- and SBP-treated groups formed a nodule by mineralization, unlike the pre-osteoblast group. In addition, the mineralization in MC3T3–E1 cells showed that treatment with SBP significantly increased dose-dependently at 14 days (Figure 4A). Stained calcium deposits were quantified by measuring absorbance after dissolving with 10% cetylpyridinium chloride. The calcium deposits in the SBP-treated group significantly increased by up to 123% at 500 μg/mL treatment (Figure 4B). These results indicate that the treatment with SBP led to an accelerated bone mineralization effect and may induce early- and late-stage osteoblast differentiation factors.

### 3.5. Effect of SB and SBP on the Expression of Genes Involved in Osteoblast Differentiation

**Figure 5:**
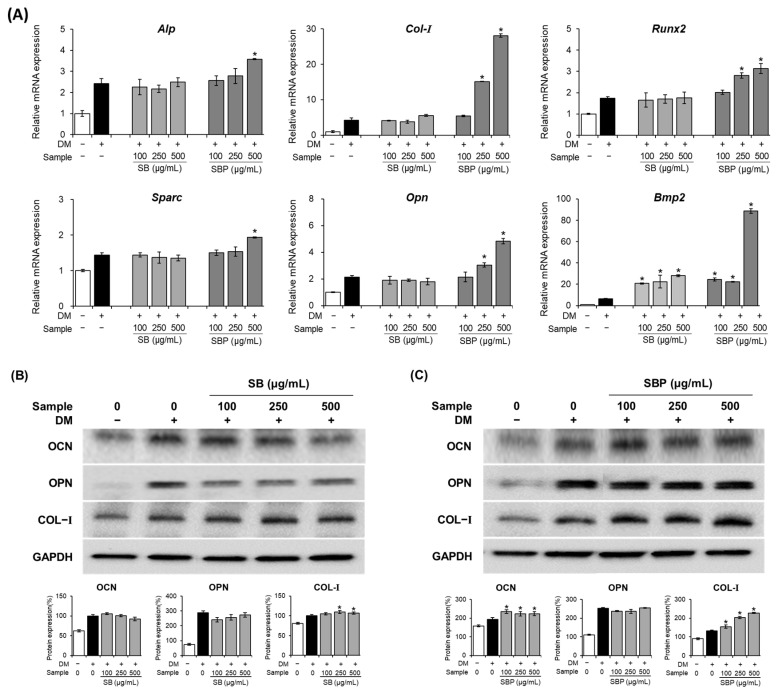
Effects of SB and SBP extracts on osteoblast differentiation in MC3T3–E1 cells. Cells were treated with or without differentiation media, with the extracts, for 14 days. (**A**) The relative mRNA expression level of osteoblast markers (*Alp*, *Runx2*, *Col–I*, *Sparc*, *Opn,* and *Bmp–2*) determined by qPCR and (**B**,**C**) the protein level of the post–differentiation markers (OCN, OPN, COL–I) assessed by Western blot. Marker expression quantifications were normalized using GAPDH and presented as fold change or percentage. Data are expressed as mean ± SD. * *p* < 0.05 compared to the DM only-treated group by Student’s *t*-test. DM, differentiation media; SB, Sword bean; SBP, Sword bean pod.

To gain further insights into the molecular mechanisms of osteoblast differentiation induced by SB and SBP, the expression of several osteoblast markers was examined by real-time PCR and Western blot on days 7 and 14 (Figure 5).

The mRNA expression levels of *Alp*, *Col–I*, *Runx2*, *Sparc*, *Bmp2*, and *Opn* were upregulated by SBP treatment (Figure 5A). The ALP gene, which aids osteoblast differentiation by transporting inorganic phosphoric acid, helping calcium phosphate deposition, and creating conditions for calcification of the extracellular matrix, increased significantly only at SBP 500 μg/mL. *Col–I* plays a vital role in inducing bone remodeling and formation. When treated with SB and SBP, *Col–I* expression was markedly elevated compared to the osteoblast group. The transcription factors *Runx2* and *Sparc* are essential in osteoblast differentiation and bone formation. The mRNA expression level of *Runx2* and *Sparc* increased by 1.7- and 1.4-fold, respectively, upon SBP treatment compared to the osteoblast groups. In addition, the expression levels of *Bmp2* in the SB and SBP treatment groups were significantly increased by up to 4- and 14-fold, respectively. *Opn*, a post-differentiation marker, increases expression during osteoblast differentiation and promotes mineralization. *Opn* mRNA expression showed patterns similar to other osteoblast genes, induced by the treatment of SB and SBP.

Compared with the pre-osteoblast group, the protein expression levels of OCN, OPN, and COL–I were regulated by the SB or SBP group (Figure 5B,C). Compared to osteoblasts, the protein levels of OCN and COL-I increased in the SBP-treated group and tended to increase dose-dependently. On the other hand, in the SB-treated group, there was a significant difference only in the protein level of COL–I, and there was no significant difference in OCN and OPN.

### 3.6. Effect of SB and SBP on the Expression of Genes Involved in the BMP2/SMAD/RUNX2 Axis

**Figure 6:**
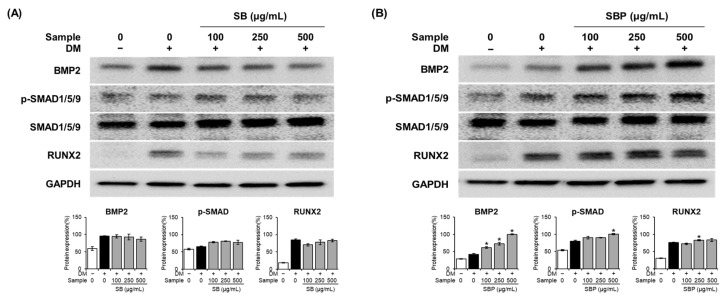
Effects of SB (**A**) and SBP (**B**) on the BMP2–Smad1/5/9 signaling pathway. Cells were treated with or without differentiation media, with extracts, for 7 days. (**A**,**B**) The protein levels of BMP2, p–SMAD1/5/9, SMAD1/5/9, and RUNX2. BMP2 and RUNX2 expression quantification were normalized using GAPDH. p-SMAD1/5/9 expression was normalized to total SMAD1/5/9. Data are expressed as mean ± SD. * *p* < 0.05 compared to the DM only-treated group by Student’s *t*-test. DM, differentiation media; SB, Sword bean; SBP, Sword bean pod.

The BMP signaling pathway is reported to play a major role in regulating osteoblastic differentiation. We evaluated the effects of SB and SBP extracts on the activation of BMP2 and p-Smad1/5/9 signaling molecules. Protein levels of BMP2 increased significantly by up to 2.4 times in the SBP-treated group compared to the osteoblast group and increased dose-dependently (Figure 6). The levels of phosphorylated SMAD1/5/9 increased significantly in the SBP-treated group than in the SB-treated group. Additionally, the expression level of the transcription factor RUNX2, the primary downstream target of BMPs, was significantly elevated upon SBP treatment. Together, these results indicate that SBP promoted osteoblast differentiation through BMP2-SMAD1/5/9 signaling and had a stronger effect than SB.

## 4. Discussion

Osteoporosis is a systemic skeletal disease that causes bones to become thin and brittle due to decreased bone mass and loss of bone tissue. Estrogen, calcitonin, and bisphosphonates are bone resorption inhibitors commonly used in clinical treatments for osteoporosis. These treatments aim to prevent bone fractures and preserve bone mass by blocking bone resorption. Nevertheless, they only have a relatively small impact on bone mineral loss and recovery, about 2% annually [37]. Given that osteoblasts are the main drivers of new bone formation, substances that stimulate osteoblast proliferation or differentiation can enhance osteogenesis [1]. Hence, searching for novel materials that can stimulate bone formation has become a pressing need to treat osteoporosis. Recently, studies on various natural materials with such effects have been reported. SB is abundant in phytochemicals with various physiological functions, but its impact on promoting the proliferation and differentiation of osteoblastic cells has not yet been determined. Furthermore, SBP is expected to perform better than SB since it contains a higher content of multiple bioactive ingredients, such as citric acid and proline. Proline, which occurs during osteoblast differentiation and bone formation, is essential [38]. Shen et al. [33] used gene editing tools to delete the proline transporter in osteoblast cells of mice and as a result, proline production was inhibited and bone formation was found to be reduced. These results demonstrate the importance of proline in the development of osteoblast cells and the synthesis of bone formation products. Moreover, citric acid is known to improve bone density and inhibit bone contraction by inhibiting the reabsorption of calcium into the bone by its chelating potential [34]. Citric acid is used as a current treatment for osteoporosis, as it increases bone density [39,40,41]. SB and SBP contain various polyphenol components, including tannin, sinapic acid, and pyrogallol [23]. It is well-known that polyphenol ingredients can be used to treat osteoporosis by promoting the proliferation of osteoblasts due to their antioxidant properties [42]. Tannin has been found to enhance osteogenesis and angiogenesis, as evidenced by immunohistochemical staining for OCN and vascular endothelial growth factor [43]. Furthermore, treatment with tannic acid alone or in combination with Pamidronate (PAM), a medication used to treat osteoporosis, was more effective in promoting osteoblast differentiation than both the control group and the PAM treatment group [36]. Sinapic acid is recognized as a potent antioxidant with osteogenic properties. Sadhasivam et al. [35] discovered that zoledronic acid decreased osteoblast viability and inhibited differentiation. Conversely, treatment with sinapic acid led to an increase in the expression levels of osteoblast marker genes, including Runx2, Col-I, and ALP. Sinapic acid also promoted osteoblast differentiation and mineralization by enhancing calcium deposition [35]. Moreover, Sinapic acid-loaded chitosan scaffolds are used for bone regeneration [44] and phenolic compounds have the potential to prevent bone defects by modulating osteoblast activity [45].

Thus, this study aimed to investigate the effects of SB and SBP on the differentiation of MC3T3–E1 cells towards osteoblasts and to explore the underlying mechanisms involved using an in vitro model.

As the enhanced proliferation of osteoblast cells is a crucial factor in promoting bone formation, we examined the impact of SB and SBP on cell viability. We observed that 100, 250, and 500 μg/mL of both SB and SBP exhibited no toxic effects and increased cell proliferation in a dose-dependent manner in MC3T3–E1 cells. These observations are consistent with our previous studies [27], which found that doses of 10–200 μg/mL of SB and SBP had non-cytotoxic effects on the viability of cells in C3H10T1/2 cells. We also observed that the proliferation rate varied depending on the treatment time, but an increase in proliferation rate with concentration was always observed. Therefore, SB and SBP stimulated cell proliferation and were non-cytotoxic toward MC3T3–E1 cells.

We show that SBP promotes the proliferation and differentiation of osteoblasts by activating the BMP2/SMAD/RUNX2 pathway. RUNX2 promotes the transcription of various mRNAs, including *Ocn*, *Opn*, and *Alp* that induce osteoblast differentiation, maturation, and mineralization [46,47]. The absence of bone tissue or osteoblasts in RUNX2-null mice has identified RUNX2 as an essential molecule inducing osteoblast differentiation [48]. The results of our study confirmed that the mRNA and protein levels of RUNX2 increased during SBP treatment. Subsequently, we confirmed the involvement of ALP, COL–I, SPARC, OPN, and BMP2 in osteoblast differentiation, which RUNX2 regulates. We also observed an increase in the degree of mineralization and collagen synthesis. ALP, an early-stage marker for osteoblast differentiation, is an essential enzyme that induces the mineralization of substrates and upregulates osteoblast differentiation genes, including COL–I. COL–I is the most abundant protein synthesized by active osteoblasts and a major indicator of the beginning of osteoblastic differentiation [47]. In this study, ALP activity and COL–I content were significantly elevated in SBP-treated cells compared to the osteoblast group, and this effect varied with concentration. The mRNA levels of Alp and *Col–I* showed a significant increase only upon SBP treatment, and the protein level of COL–I was significantly increased by the SB and SBP treatment. In particular, it increased by 1.7-fold in the 500 µg/mL SBP-treated group. In addition, matrix mineralization was higher in the SBP-treated group than in the osteoblast group and showed no significant difference in SB. These data indicated that SBP could promote cell differentiation in MC3T3-E1 cells, not SB.

Unlike other tissues, bones are mostly inorganic, and because the entire organic material is collagen, collagen plays a vital role in the structure and function of bone tissue, and changes in its functions are also related to osteoporosis [49]. Non-collagenous proteins (NCPs) comprise 10–15% of bone proteins and are mainly involved in mineralization [50]. Recent experiments in mice have shown that deletion of SPARC, one of the NCPs, causes osteopenia due to low bone turnover, which is an apparent defect in both osteoblast and osteoblast activity [49]. In addition, the level of SPARC decreases when RUNX2 is silenced [51]. OPN and OCN are also included in bone-specific NCPs and form complexes to regulate bone mineralization through a strong affinity for hydroxyapatite [52]. Without these two proteins, the complex is destroyed, resulting in dramatic personality loss. Both OPN and OCN are regulated by RUNX2, which acts as a transcription factor [53,54]. Our results showed that the mRNA levels of *Sparc* and *Opn* increased with the increase in *Runx2* induced by SBP treatment, and there was no significant increase by SB. In addition, we observed that the protein levels of OCN and OPN were also elevated. This suggests the induction and promotion of bone matrix mineralization, taken together with the COL–I and ARS results, it is confirmed that SBP promotes mineralization.

The expression of RUNX2 and the differentiation of osteoblasts are tightly controlled by several extracellular proteins and intracellular signaling pathways [46]. In particular, BMP2 induces RUNX2 expression by activating intracellular proteins such as SMAD1/5/9. According to the results of our study, SBP treatment induces RUNX2 expression through BMP2-mediated SMAD1/5/9 signal transduction, while SB treatment showed no significant effect.

In addition, the MAPK signaling pathway involves RUNX2 as a downstream molecule, and BMP2 probably plays a role in the MAPK signaling pathway during the stimulation of differentiation by SBP. MAPKs refer to a group of enzymes known as serine/threonine kinases, which serve vital functions in numerous cellular processes, including cell growth, differentiation, and inflammation. They can be activated by external stimuli such as growth factors, cytokines, and stress and are involved in transmitting signals from the cell membrane to the nucleus, where they regulate gene expression and cellular responses [3]. Recent studies have reported that three members of the MAPK family, extracellular signal-regulated kinase (ERK), c-Jun N-amino-terminal kinase (JNK), and p38, are activated by BMP2/SMAD-dependent signals and could affect osteoblast differentiation. It has also been demonstrated that activation of the MAPK and Erk1/2 signaling pathway played a key role in osteogenesis and bone homeostasis by upregulating bone formation-related gene expressions [5,12,55,56,57]. Therefore, it is necessary to determine whether the MAPK signaling induced osteoblast differentiation upon treatment with SB and SBP.

**Figure 7:**
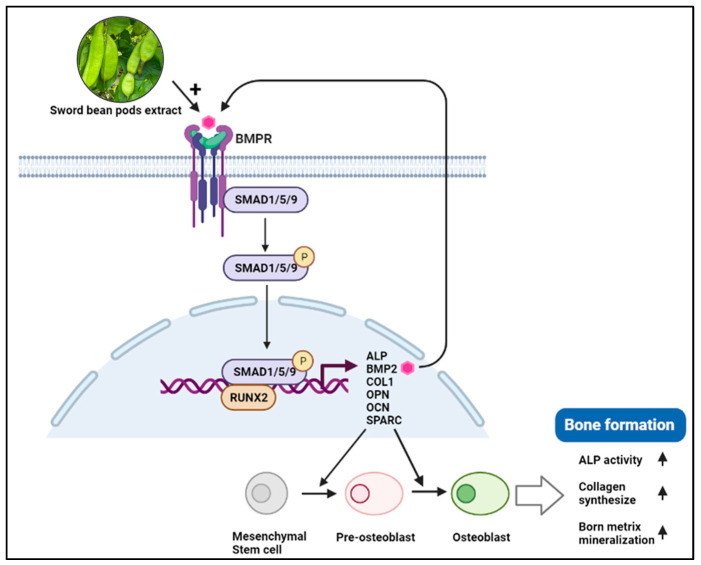
Schematic representation of SBP effects on BMP2-Smad1/5/9-Runx2 signaling pathway. BMP (Bone morphogenetic protein2), BMPR (BMP receptor), RUNX2 (Runt-related transcription factor 2), OPN (Osteopontin), OCN (Osteocalcin), SPARC (Secreted protein acidic and cysteine-rich), ALP (Alkaline phosphatase), COL-I (Type I collagen).

Taken together, our results suggest that SBP treatment increased the expression of ALP, COL–I, OPN, OCN, SPARC, and RUNX2, inducing osteoblast differentiation through BMP2/SMAD/RUNX2 pathway. The SBP treatment promoted a more robust osteoblast differentiation, mineralization, and collagen synthesis than the SB treatment (Figure 7).

## 5. Conclusions

This study investigated whether SB and SBP extracts ultimately affect osteoblast differentiation by regulating bone-specific transcription factors and BMP2/SMAD/RUNX2 signaling to influence marker gene and protein expression. Using the osteoblast cell line, MC3T3-E1, we found that SB and SBP regulated BMP2 signaling by regulating BMP2 expression and the downstream regulator SMAD1/5/9 activity. Elevated BMP2 and SMAD 1/5/9 expression caused an increase in bone-specific transcription factor RUNX2 and other bone marker genes and proteins (ALP, COL-I, OPN, OCN, SPARC). These findings suggest that SB and SBP can induce osteoblast differentiation by increasing the BMP2/SMAD/RUNX2 signaling pathway, and in particular, SBP promotes osteoblast differentiation, mineralization, and collagen synthesis better than SB. In order to elucidate the exact mechanism of bone health associated with SBP, follow-up studies on active components, animal experiments, and clinical studies should be conducted in the future.

## Figures and Tables

**Table 1:** Primer sequences.

Gene	Primer Sequences (5′→3′)	
ALP	Forward	AACCCAGACACAAGCATTCC
	Reverse	GAGAGCGAAGGGTCAGTCAG
COLI	Forward	CAAGATGTGCCACTCTGACT
	Reverse	TCTGACCTGTCTCCATGTTG
Runx2	Forward	ACTCTTCTGGAGCCGTTTATG
	Reverse	GTGAATCTGGCCATGTTTGTG
SPARC	Forward	AAACATGGCAAGGTGTGTGA
	Reverse	TGCATGGTCCGATGTAGTC
OPN	Forward	AGCAAGAAACTCTTCCAAGCAA
	Reverse	GTGAGATTCGTCAGATTCATCCG
BMP2	Forward	ACACAGCTGGTCACAGATAAG
	Reverse	CTTCCGCTGTTTGTGTTTGG
GAPDH	Forward	GTCAAGGCTGAGAACGGGAA
	Reverse	AAATGAGCCCCAGCCTTCTC

## Data Availability

Not applicable.

## Supplementary Materials

The following supporting information can be downloaded at: https://www.mdpi.com/article/10.3390/nu15204372/s1, Figure S1. The sword bean (A) and immature sword bean pods (B).
