# Supplementary figures and images for: Sword Bean (Canavalia gladiata) Pods Induce Differentiation in MC3T3-E1 Osteoblast Cells by Activating the BMP2/SMAD/RUNX2 Pathway
Source: Nutrients. 2023 Oct 16;15(20):4372. doi: 10.3390/nu15204372 (PMC10610144; doi:10.3390/nu15204372)

**(A)**

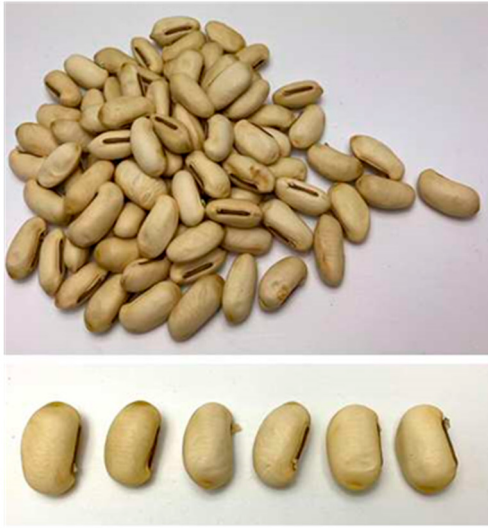

**(B)**

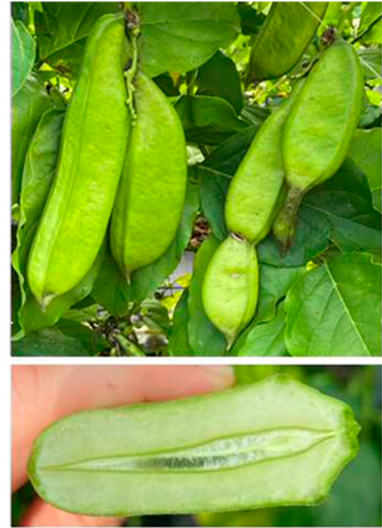

**Figure S1.** The sword bean (A) and immature sword bean pods (B).

Supplement: Supplementary file 1 [file nutrients-15-04372-s001.zip › nutrients-2601609-supplementary.pdf]
